# Some Families of Jensen-like Inequalities with Application to Information Theory

**DOI:** 10.3390/e25050752

**Published:** 2023-05-04

**Authors:** Neri Merhav

**Affiliations:** The Viterbi Faculty of Electrical and Computer Engineering, Technion—Israel Institute of Technology, Technion City, Haifa 3200003, Israel; merhav@ee.technion.ac.il; Tel.: +972-4-8294737

**Keywords:** Jensen’s inequality, convex function, concave function, entropy, capacity, moment-generating function, cumulant-generating function

## Abstract

It is well known that the traditional Jensen inequality is proved by lower bounding the given convex function, f(x), by the tangential affine function that passes through the point (E{X},f(E{X})), where E{X} is the expectation of the random variable *X*. While this tangential affine function yields the tightest lower bound among all lower bounds induced by affine functions that are tangential to *f*, it turns out that when the function *f* is just part of a more complicated expression whose expectation is to be bounded, the tightest lower bound might belong to a tangential affine function that passes through a point different than (E{X},f(E{X})). In this paper, we take advantage of this observation by optimizing the point of tangency with regard to the specific given expression in a variety of cases and thereby derive several families of inequalities, henceforth referred to as “Jensen-like” inequalities, which are new to the best knowledge of the author. The degree of tightness and the potential usefulness of these inequalities is demonstrated in several application examples related to information theory.

In memory of Jacob Ziv, a shining star in the sky of information theory, whose legacy as a researcher will continue to inspire me and many others for years to come.

## 1. Introduction

As is well known, the Jensen inequality is one of the most fundamental and useful mathematical tools in a variety of fields, including information theory. Interestingly, it includes many other very well-known inequalities, which are important on their own, as special cases. Among many examples, we mention the Shwartz–Cauchy inequality (which in turn supports uncertainty principles and the Cramér–Rao bound), the Lyapunov inequality, the Hölder inequality, and the inequalities among the harmonic, geometric and arithmetic means. In the field of information theory, the Jensen inequality stands at the basis of the information inequality (i.e., the non-negativity of the relative entropy), the data processing inequality (which in turn leads to the Fano inequality), and the inequality between conditional and unconditional entropies. Moreover, it plays a central role in support of the derivation of single-letter formulas in Shannon theory and in the theory of maximum entropy under moment constraints (see, for example, Chapter 12 of [1]).

During the last two decades, there have been many research efforts around Jensen’s inequality, which included refinements [2,3,4,5], variations [6,7,8], improvements [9,10,11], and extensions [12], just to name a few. There have also many derivations of reversed versions of the Jensen inequality. For a non-exhaustive list of works, see, e.g., ref. [13] for mixtures of exponential families, refs. [14,15,16,17] for global bounds on the difference between the two sides of Jensen’s inequality, ref. [18] for functions of self-adjoint operators in Hibert spaces, refs. [19,20] for inequalities via Green functions, refs. [21,22] for inequalities via Chebychev and Chernoff bounds, ref. [23] for quantum Simpson’s and quantum Newton’s inequalities, and ref. [24] for new quantum Hermite–Hadamard-like inequalities. In most of them, the derived inequalities are exemplified in many applications, for instance, useful relationships between arithmetic and geometric means, converse bounds on the entropy, the relative entropy, as well as the more general *f*-divergence, converse forms of the Hölder inequality, and so on. In many of these works, the main results are given in the form of an upper bound on the difference, E{f(X)}−f(E{X}), where *f* is a convex function, E{·} is the expectation operator, and *X* is the random variable. However, those bounds depend mostly on global parameters associated with *f*, for example, its range and domain, but not particularly on the underlying probability function (probability density function in the continuous case, or probability mass function in the discrete case), of *X*. For one thing, a desirable property of a reverse Jensen inequality would be that it is tight when *X* is well concentrated in the vicinity of its mean, just like the same well-known property of the ordinary Jensen inequality. In [22], there is an attempt to address this issue.

This paper revisits the Jensen inequality from a completely different angle. It is not meant to be another improvement of earlier bounds in an existing line of work. It is meant to propose a different approach for generating useful inequalities in the spirit of Jensen’s inequality. It is based on the following simple observation, which is rooted in the proof of Jensen’s inequality: The given convex function, f(x), is lower bounded by the tangential affine function, $\ell \left(x\right)=f\left(a\right)+f^{\prime }\left(a\right)\left(x-a\right)$, where *a* is an arbitrary number in the domain of *x* and $f^{\prime }\left(a\right)$ is the derivative of *f* at x=a (provided that *f* is differentiable at x=a). By selecting a=E{X} and taking expectations of both sides of the inequality, f(X)≥ℓ(X), the Jensen inequality is readily proved. The point to be remembered is that here, $a_{*}=E\left\{X\right\}$ is the *optimal choice of a* in the sense of maximizing E{ℓ(X)} over all possible values of *a*, thus yielding the tightest lower bound within this class of lower bounds on E{f(X)}. The optimal choice of *a*, however, might be different than E{X} when the function f(X) is only a part of a more complicated expression whose expectation is to be lower bounded. For example, one might be interested in lower bounding E{g[f(X)]}, where *g* is a monotonically non-decreasing function, or E{f(X)g(X)}, where *g* is a non-negative and/or convex function, or a combination of both, etc.

To demonstrate this fact, consider the example (to be treated in detail in Section 2) of lower bounding E{f(X)g(X)}, where *g* is a non-negative function. In this case,

(1) $$E\left\{f\left(X\right)g\left(X\right)\right\}\geq E\{\left[f\left(a\right)+f^{\prime }\left(a\right)\left(X-a\right)\right]g\left(X\right)\},$$

and by maximizing the right-hand side (r.h.s.) over *a*, we easily obtain that the optimal choice of *a* here is $a_{*}=E\left\{Xg\left(X\right)\right\}/E\left\{g\left(X\right)\right\}$, yielding the inequality,

(2) $$E\left\{f\left(X\right)g\left(X\right)\right\}\geq f\left(\frac{E\{Xg(X)\}}{E\{g(X)\}}\right)\cdot E\left\{g\left(X\right)\right\},$$

which is useful as long as *g* is such that we can easily calculate both E{g(X)} and E{Xg(X)}. While this particular inequality could have been obtained also by applying the (ordinary) Jensen inequality, E{f(X)}≥f(E{X}), with respect to (with respect to) the density, $\tilde{p}\left(x\right)=p\left(x\right)g\left(x\right)/\int_{-\infty }^{\infty }p\left(x^{\prime }\right)g\left(x^{\prime }\right)dx^{\prime }$, we will see in the sequel also various examples of inequalities with no apparent simple interpretations such as this. We henceforth refer to these classes of inequalities as *Jensen-like inequalities,* since they are derived using the same general idea that underlies the proof the classical Jensen inequality. We will also demonstrate the usefulness of these inequalities in information theory.

Our contributions, in this work, have the following features:

1. In many cases (such as the one above), the optimal value of the parameter(s) (e.g., the parameter *a* in the above discussion) can be found in closed form. In other cases, the resulting expressions may not lend themselves to closed-form optimization, and then we have two possibilities: (i) carry out the optimization numerically, and (ii) select an arbitrary choice of *a* and obtain a valid lower bound, bearing in mind that an educated guess can potentially result in a good bound.
2. Our inequalities provide two types of bounds: (i) bounds that require the calculation of the first two moments (or equivalently, the first two cumulants) of *X*, and (ii) bounds that require the calculation of the moment-generating function (MGF) of *X* and its derivative, or equivalently, the cumulant-generating function (CGF) of *X* and its derivative. All these types of moments are often easily calculable in closed form, especially in situations where *X* is given by the sum of independent and identically distributed (i.i.d.) random variables, which is frequently encountered in information–theoretic applications.
3. Most of our derivations extend to convex functions of more than one variable.
4. The classes of Jensen-like inequalities that we consider allow enough flexibility to obtain derivations of lower bounds on functions that are not necessarily convex, and even for some concave functions, and thereby open the door for another route to reverse Jensen inequalities. This can be accomplished by representing the given function in one of the categories discussed (e.g., a product of a convex function and a non-negaive function, a product of two non-negative convex functions, a composition of a monotone function and a convex function, etc.).
5. We demonstrate the utility of the Jensen-like inequalities in several examples of information–theoretic relevance. We also display numerical results that exemplify the degree of tightness of these bounds.
6. Our Jensen-like inequalities have the desirable property of becoming tighter as *X* becomes more and more concentrated around its mean, just like the ordinary Jensen inequality.
7. Throughout the paper, we confine ourselves to lower bounds on expectations of expressions that include a convex function *f*, but it should be understood that they all continue to apply also if *f* is concave and the inequalities are reversed.
8. It should be understood that the classes of Jensen-like inequalities that we derive in this work are just examples that demonstrate the basic underlying idea of optimizing the point of tangency to the given convex function for the specific expression at hand. It is conceivable that the same idea can be applied to many more situations of theoretical and practical interest.

In all forthcoming derivations, it will be assumed that the convex functions involved are weakly convex and differentiable. In other words, we will rely on the well-known fact that a differentiable convex function, f(x), is nowhere below the supporting line, $\ell \left(x\right)=f\left(a\right)+f^{\prime }\left(a\right)\left(x-a\right)$, for every value of the parameter *a* in the domain of the independent variable, *x* [25] (p. 69, eq. (3.2)). In order to show that the point of zero-derivative of the lower bound (w.r.t. *a*) indeed yields a maximum (and not a minimum, etc.) of the lower bound, we will need to further assume that *f* is twice differentiable, but such an assumption will not limit the applicability of the claimed lower bound, because the lower bound applies to any value of *a*, including the point of zero-derivative, even if this point cannot be proved to yield the maximum of the lower bound using the standard methods. Similar comments apply when the lower bound will depend on more than one parameter.

In the remaining part of this article, each section is devoted to a different class of Jensen-like inequalities, which corresponds to a different form of an expression that includes the convex function, *f*.

## 2. A Product of a Convex Function and a Non-Negative Function

In this section, we focus on lower bounding expressions of the form E{f(X)g(X)}, where *f* is convex and *g* is non-negative. Indeed, let f:R→R be a convex function and let $g:R\to R^{+}$ be a non-negative function. Then, for any a∈R,

(3)E{f(X)g(X)}≥E{[f(a)+f′(a)(X−a)]g(X)}(4)=[f(a)−af′(a)]E{g(X)}+f′(a)E{Xg(X)}.

To find the value of *a* that maximizes the r.h.s., we equate the derivative to zero and obtain:

(5) $$\left[f^{\prime }\left(a\right)-f^{\prime }\left(a\right)-af^{\prime \prime }\left(a\right)\right]E\left\{g\left(X\right)\right\}+f^{\prime \prime }\left(a\right)E\left\{Xg\left(X\right)\right\}=0$$

or equivalently,

(6) $$f^{\prime \prime }\left(a\right)\left[E\left\{Xg\left(X\right)\right\}-aE\left\{g\left(X\right)\right\}\right]=0,$$

whose solution is readily obtained as

(7) $$a=a_{*}\overset{▵}{=}\frac{E\{Xg(X)\}}{E\{g(X)\}},$$

and it is easy to verify that the second derivative at $a=a_{*}$ is $-f^{\prime \prime }\left(a_{*}\right)E\left\{g\left(X\right)\right\}<0$, which means that it is a maximum (at least a local one). The resulting lower bound on E{f(X)g(X)} is then given by

(8) $$E\left\{f\left(X\right)g\left(X\right)\right\}\geq f\left(\frac{E\{Xg(X)\}}{E\{g(X)\}}\right)\cdot E\left\{g\left(X\right)\right\}.$$

This result extends straightforwardly to the case where *X* is a vector provided that *f* is jointly convex and differentiable in all components of *X*. In particular, it extends to the case where *f* and *g* act as different random variables, *X* and *Y*, with a joint distribution:

(9) $$E\left\{f\left(X\right)g\left(Y\right)\right\}\geq f\left(\frac{E\{Xg(Y)\}}{E\{g(Y)\}}\right)\cdot E\left\{g\left(Y\right)\right\}.$$

We next consider several examples.

**Example 1.**  *Let f(x)=−lnx and g(x)=x, x>0. Applying Inequality (8),*

(10) $$E\left\{-X\ln X\right\}\geq -E\left\{X\right\}\cdot \ln\frac{E\{X^{2}\}}{E\{X\}}=-E\left\{X\right\}\cdot \ln\left(E\left\{X\right\}\right)-E\left\{X\right\}\cdot \ln\left(1+\frac{Var\{X\}}{{\left[E\left\{X\right\}\right]}^{2}}\right).$$

*Note that the function −xlnx is concave, rather than convex, yet we have here a lower bound (rather than an upper bound) to its expectation, namely, a reversed Jensen inequality. The first term on the right-most side is the (ordinary) Jensen upper bound on E{−XlnX}, and the second term is the gap, which depends not only on the expectation of X but also on its variance, which manifests the fluctuations around E{X}. Clearly, if Var{X}=0, the second term vanishes, which makes sense, because when X is a degenerated random variable, Jensen’s inequality is achieved with equality and there is no gap. This inequality has an immediate application for obtaining a lower bound to the expectation of the empirical entropy of a sequence drawn by a memoryless source, which is relevant in the context of universal source coding [26]. Each term of the empirical entropy is of the form −XlnX, where X=N(u)/N, N(u) is the number of occurrences of a letter u in a randomly drawn N-tuple from a memoryless source, P, with a finite alphabet, U. Clearly, each N(u) is a binomial random variable with N trials and probability of success, P(u). In this case, E{X}=P(u) and Var{X}=P(u)[1−P(u)]/N. Thus, denoting the entropy and the empirical entropy, respectively, by*

(11) $$\begin{array}{ccc} H & = & -\sum_{u\in U}P\left(u\right)\ln P\left(u\right) \end{array}$$

(12) $$\begin{array}{ccc} \hat{H} & = & -\sum_{u\in U}\frac{N(u)}{N}\ln\left(\frac{N(u)}{N}\right), \end{array}$$

*with the convention that $0\ln0\overset{▵}{=}0$, we have:*

E{H^}≥−∑u∈UP(u)lnP(u)−∑u∈UP(u)ln1+P(u)[1−P(u)]/NP2(u)=H−∑u∈UP(u)ln1+1−P(u)NP(u)≥H−∑u∈UP(u)·1−P(u)NP(u)=H−1N∑u∈U[1−P(u)](13)=H−|U|−1N,

*where |U| is the cardinality of U. The use of the ordinary Jensen inequality yields an upper bound rather than a lower bound, $E\{\hat{H}\}\leq H$. We conclude that the expected empirical entropy, $E\{\hat{H}\}$, is sandwiched between H and H−(|U|−1)/N, which is reasonable because the variance of the empirical probabilities, N(u)/N, decays at the rate of 1/N.*

**Example 2.**  *Let s and t be two real numbers whose difference, s−t, is either negative or larger than unity. Now, let $g\left(x\right)=x^{t}$, and $f\left(x\right)=x^{s-t}$. Then,*

E{Xs}=E{XtXs−t}≥E{Xt+1}E{Xt}s−t·E{Xt}(14)=(E{Xt+1})s−t(E{Xt})s−t−1.

*In particular, for t=1 and s∉(1,2), this becomes*

(15) $$E\left\{X^{s}\right\}\geq \frac{{\left(E\left\{X^{2}\right\}\right)}^{s-1}}{{\left(E\left\{X\right\}\right)}^{s-2}}={\left[E\left\{X\right\}\right]}^{s}\cdot {\left(1+\frac{Var\{X\}}{{\left[E\left\{X\right\}\right]}^{2}}\right)}^{s-1}$$

*which is, once again, a bound that depends only on the first two moments of X. For s∈(0,1), the function $x^{s}$ is concave, and so, this is a reversed version of the Jensen inequality. For s≤0 and s≥2, the function $x^{s}$ is convex, and so, this is an improved version of the Jensen inequality: While the first factor, ${\left[E\left\{X\right\}\right]}^{s}$, corresponds to the ordinary Jensen inequality, the second factor expresses the improvement, which depends on the relative fluctuation term, $Var\left\{X\right\}/{\left[E\left\{X\right\}\right]}^{2}$. The degree of improvement depends, of course, on the variance of X. If the variance vanishes, there is nothing to improve because the ordinary Jensen inequality becomes an equality. On the other hand, the larger the variance, the larger the gap between the ordinary Jensen bound, ${\left[E\left\{X\right\}\right]}^{s}$, and the improved one. Accordingly, this also demonstrates the role of the optimization of the parameter a as opposed to the default choice of a=E{X} of the ordinary Jensen inequality.*

*To particularize this example even further, consider the problem of randomized guessing under a distribution Q (see, e.g., *[27]* and many references therein). Then, the probability of a single success in guessing a discrete alphabet random variable, X, given that we know that X=x (but not the guesser), is Q(x). In sequential guessing until the first success, the number of guesses, G, is a geometric RV with parameter p=Q(x), whose mean and variance are 1/p and $\left(1-p\right)/p^{2}$, respectively. For s∈(1,2),*

(16) $$E\left\{G^{s}\right\}\geq {\left(\frac{1}{p}\right)}^{s}\cdot {\left(1+\frac{\left(1-p\right)/p^{2}}{1/p^{2}}\right)}^{s-1}=\frac{{\left(2-p\right)}^{s-1}}{p^{s}}=\frac{{\left[2-Q\left(x\right)\right]}^{s-1}}{{\left[Q\left(x\right)\right]}^{s}}.$$

**Example 3.**  *Let f be an arbitrary convex function and let $g\left(x\right)=e^{sx}$, where s is a given real number. Then, Inequality (8) becomes:*

(17) $$E\left\{f\left(X\right)e^{sX}\right\}\geq f\left(\psi^{\prime }\left(s\right)\right)\cdot e^{\psi (s)}$$

*where*

(18) $$\psi \left(s\right)=\ln E\left\{e^{sX}\right\}$$

*is the CGF of X and $\psi^{\prime }\left(s\right)$ is its derivative. This gives a lower bound in terms of the CGF of X and its derivative. The ordinary Jensen inequality is obtained as the special case of s=0, where ψ(0)=0 and $\psi^{\prime }\left(0\right)=E\left\{X\right\}$.*

## 3. A Composition of a Monotone Function and a Convex Function

Another family of Jensen-like inequalities corresponds to the need to lower bound an expression of the form E{g[f(X)]}, where *f* is convex as before and *g* is a monotonically non-decreasing function. The general idea is to carry out the optimization of the r.h.s. of the following inequality.

(19) $$E\left\{g\left[f\left(X\right)\right]\right\}\geq \underset{a}{\sup}E\left\{g\left[f\left(a\right)+f^{\prime }\left(a\right)\left(X-a\right)\right]\right\}.$$

In the important special case where $g\left(x\right)=e^{x}$, we have:

E{ef(X)}≥supaE{ef(a)+f′(a)(X−a)}=supaef(a)−af′(a)E{eXf′(a)}(20)=expsupa{f(a)−af′(a)+ψ[f′(a)]},

where ψ(·) is again the CGF of *X*. The optimal value, $a_{*}$, of *a*, is the solution to the equation obtained by equating the derivative of the exponent to zero, i.e.,

(21) $$\psi^{\prime }\left[f^{\prime }\left(a_{*}\right)\right]=a_{*},\;\;\mathrm{provided}\;\mathrm{that}\;\;f^{\prime \prime }\left(a_{*}\right)\psi^{\prime \prime }\left[f^{\prime }\left(a_{*}\right)\right]<1,$$

where $\psi^{\prime }\left(\cdot \right)$ and $\psi^{\prime \prime }\left(\cdot \right)$ are the first and the second derivatives of ψ(·), respectively.

**Example 4.**  *Consider the case where $f\left(x\right)=sx^{2}/2$ and $X∼N(\mu ,\sigma^{2})$, where $\sigma^{2}<1/s$, as otherwise, $E\{e^{sX^{2}}\}=\infty$. In this case, the condition $f^{\prime \prime }\left(a_{*}\right)\psi^{\prime \prime }\left[f^{\prime }\left(a_{*}\right)\right]<1$ is equivalent to $\sigma^{2}<1/s$, and we have $f^{\prime }\left(a\right)=sa$, $\psi \left(t\right)=\mu t+\sigma^{2}t^{2}/2$, and so, $\psi^{\prime }\left(t\right)=\mu +\sigma^{2}t$, which means that $\psi^{\prime }\left[f^{\prime }\left(a\right)\right]=\mu +\sigma^{2}sa$. The equation for the optimal a becomes then*

(22) $$\mu +\sigma^{2}sa=a,$$

*whose solution is*

(23) $$a=a_{*}\overset{▵}{=}\frac{\mu }{1-\sigma^{2}s},$$

*which yields*

(24) $$E\left\{e^{sX^{2}/2}\right\}\geq \exp\left\{sa_{*}^{2}/2-sa_{*}^{2}+\mu sa_{*}+\sigma^{2}s^{2}a_{*}^{2}/2\right\}=\exp\left\{\frac{\mu^{2}s}{2(1-\sigma^{2}s)}\right\}.$$

*The ordinary Jensen inequality yields*

(25) $$E\left\{e^{sX^{2}/2}\right\}\geq \exp\left\{sE\{X^{2}\}/2\right\}=e^{s(\mu^{2}+\sigma^{2})/2},$$

*which does not capture the singularity at $s=1/\sigma^{2}$. The exact calculation yields*

(26) $$E\left\{e^{sX^{2}/2}\right\}=\frac{1}{\sqrt{1-\sigma^{2}s}}\cdot \exp\left\{\frac{\mu^{2}s}{2(1-\sigma^{2}s)}\right\},$$

*namely, the Jensen-like bound (24) gives the correct exponential term (along with the singularity at $s=1/\sigma^{2}$) and differs from the exact quantity only in the pre-exponential factor. Once again, this demonstrates the fact that optimizing the point of tangency, a, rather than using the default value, a=E{X}, can make a significant difference.*

## 4. A Product of a Convex Function and a Monotone-Convex Composition

Yet another class of Jensen-like inequalities corresponds to lower bounding the expectation of the product of two functions, where one is convex and the other is a composition of a non-negative monotonically non-decreasing function and a convex function, i.e.,

(27) $$E\left\{h\left[f\left(X\right)\right]g\left(X\right)\right\}\geq \underset{a,b}{\sup}E\left\{h\left[f\left(a\right)+f^{\prime }\left(a\right)\left(X-a\right)\right]\cdot \left[g\left(b\right)+g^{\prime }\left(b\right)\left(X-b\right)\right]\right\},$$

where *f* and *g* are convex and *h* is monotonically non-decreasing and non-negative. For the case where $h\left(x\right)=e^{x}$, we end up with a bound that depends on the CGF of *X* and its derivative:

(28)E{ef(X)g(X)}≥Eef(a)+f′(a)(X−a)[g(b)+g′(b)(X−b)](29)=ef(a)−af′(a)EeXf′(a)[g(b)−bg′(b)+g′(b)X](30)=exp{f(a)−af′(a)+ψ[f′(a)]}{g(b)+g′(b)(ψ′[f′(a)]−b)}.

Maximizing with respect to *b* while *a* is kept fixed yields $b_{*}=\psi^{\prime }\left[f^{\prime }\left(a\right)\right]$, and we obtain:

(31) $$E\left\{e^{f(X)}g\left(X\right)\right\}\geq \underset{a}{\sup}\exp\left\{f\left(a\right)-af^{\prime }\left(a\right)+\psi \left[f^{\prime }\left(a\right)\right]\right\}\cdot g\left(\psi^{\prime }\left[f^{\prime }\left(a\right)\right]\right).$$

**Example 5.**  *Considering the case where f(x)=−lnx and g(x)=xlnx, we may obtain a reversed Jensen-like inequality, namely, a lower bound to the expectation of the concave function lnX:*

(32)E{lnX}=Ee−lnX·XlnX(33)≥supa≥0exp{−lna+1+ψ(−1/a)}·ψ′(−1/a)lnψ′(−1/a)(34)=supα≥0exp{lnα+1+ψ(−α)}ψ′(−α)lnψ′(−α)(35)=e·supα≥0αeψ(−α)ψ′(−α)lnψ′(−α)(36)=e·supα≥0αE{Xe−αX}lnE{Xe−αX}E{e−αX}.

*Defining the MGF $\phi \left(s\right)=E\left\{e^{sX}\right\}=e^{\psi (s)}$, we have:*

(37)E{lnX}≥e·supα≥0αϕ′(−α)lnψ′(−α)(38)=e·supα≥0αϕ(−α)ψ′(−α)lnψ′(−α)(39)=e·supα≥0αϕ′(−α)lnϕ′(−α)ϕ(−α).

*We obtained a lower bound in terms of the MGF and its derivative (or, equivalently, the CGF and its derivative), which is appealing in cases where X is the sum of i.i.d. random variables.*

Accordingly, we now particularize this example further by examining the case where $X=1+\sum_{i=1}^{k}Y_{i}^{2}$, with $Y_{i}∼N\left(0,\sigma^{2}\right)$, i=1,…,k, being independent random variables. The motivation of assessing an expression of the form, $E\left\{\ln\left(1+\sum_{i=1}^{k}Y_{i}^{2}\right)\right\}$, is two-fold. The first is that it is useful for bounding the ergodic capacity of the single-input, multiple-output (SIMO) channel, where $\left\{Y_{i}\right\}$ designates random channel transfer coefficients (see, e.g., [22,28,29] and references therein). The second is that it is relevant for bounding the joint differential entropy associated with the multivariate Cauchy density. Here, $\left(Y_{1},\ldots ,Y_{k}\right)$ are not Gaussian as defined above, but their multivariate Cauchy density can be represented as a continuous mixture of i.i.d. zero-mean Gaussian random variables, where the mixture is taken over all possible variances—see [22] (Example 6) for the details. In this case,

(40)ϕ(s)=Eexps1+∑i=1kYi2(41)=esE{esY2}k(42)=es(1−2sσ2)k/2,s<12σ2.

Thus,

(43) $$\psi \left(s\right)=s-\frac{k}{2}\ln\left(1-2s\sigma^{2}\right),$$

and

(44) $$\psi^{\prime }\left(s\right)=1+\frac{k\sigma^{2}}{1-2s\sigma^{2}}.$$

It follows that

(45) $$E\left\{\ln\left(1+\sum_{i=1}^{k}Y_{i}^{2}\right)\right\}\geq e\cdot \underset{\alpha \geq 0}{\sup}\left\{\frac{\alpha e^{-\alpha }}{{\left(1+2\alpha \sigma^{2}\right)}^{k/2}}\left(1+\frac{k\sigma^{2}}{1+2\alpha \sigma^{2}}\right)\ln\left(1+\frac{k\sigma^{2}}{1+2\alpha \sigma^{2}}\right)\right\}.$$

**Figure 1:**
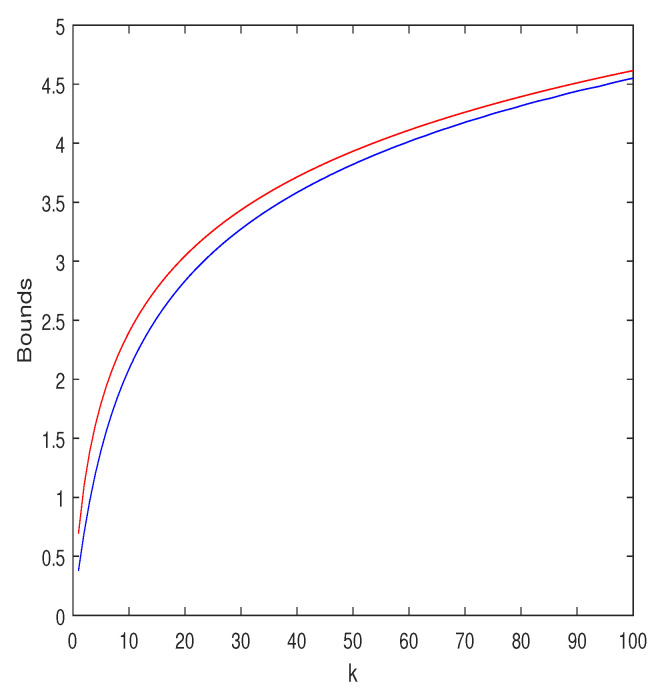
Upper and lower bounds on $E\left\{\ln\left(1+\sum_{i=1}^{k}Y_{i}^{2}\right)\right\}$, where $Y_{i}∼N\left(0,\sigma^{2}\right)$ are i.i.d., for $\sigma^{2}=1$ and k=1,2,…,100. The red curve is the upper bound, $\ln(1+k\sigma^{2})$, which is obtained by applying the ordinary Jensen inequality. The blue curve is the lower bound of Equation (45), where the search over α was carried out with a resolution of 0.001.

The Jensen upper bound, $\ln(1+k\sigma^{2})$, and the lower bound (45) are displayed in Figure 1 for $\sigma^{2}=1$ and k=1,2,…,100. As can be seen, the bounds are quite close. Interestingly, the choice $\alpha =1/(k\sigma^{2})$ yields results that are very close to those of the optimal α.

Another instance of this example is the circularly symmetric complex Gaussian channel whose signal-to-noise ratio (SNR), *Z*, is a random variable (e.g., due to fading), which is known to both the transmitter and the receiver. The capacity is given by C=E{ln(1+gZ)}, where *g* is a certain deterministic gain factor and the expectation is with respect to the randomness of *Z*. For simplicity, let us assume that *Z* is distributed exponentially, i.e.,

(46) $$p\left(z\right)=\left\{\begin{array}{cc} \theta e^{-\theta z} & z\geq 0\\ 0 & z<0 \end{array}\right.$$

where the parameter θ>0 is given. In this case,

(47) $$\phi \left(-\alpha \right)=\frac{\theta e^{-\alpha }}{\theta +g\alpha }$$

and

(48) ψ(−α)=lnθ−ln(θ+gα)−α,

and so,

(49) $$E\left\{\ln\left(1+gZ\right)\right\}\geq e\theta \cdot \underset{\alpha \geq 0}{\sup}\frac{\alpha e^{-\alpha }}{\theta +g\alpha }\cdot \left(1+\frac{g}{g+\theta \alpha }\right)\ln\left(1+\frac{g}{g+\theta \alpha }\right).$$

**Figure 2:**
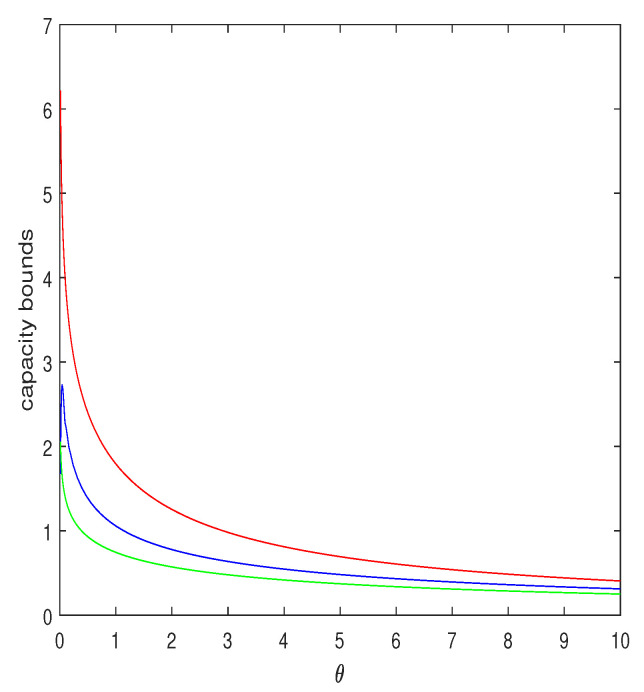
Upper and lower bounds on $E\left\{\ln(1+gZ)\right\}$, where *Z* is distributed exponentially with parameter θ, as functions of θ, for g=5. The red curve is the upper bound, ln(1+g/θ), obtained by applying the ordinary Jensen inequality. The blue curve is the lower bound of of Equation (49), where the search over α was carried out with resolution of 0.001. The green curve is the lower bound of [22] (Example 1).

In Figure 2, we plot this lower bound as a function of θ for g=5 and compare it to the Jensen upper bound, ln(1+g/θ) (red curve) and to the lower bound of [22] (Sect. 4.1, Example 1). As can be seen, the lower bound proposed here is considerably tighter, especially for small θ.

**Example 6.**  *Yet another example of this family of Jensen-like inequalities applies to obtaining a lower bound to $E\{X^{t}\}$, where t is an arbitrary real. For a given t, let s≥0 be either larger than 1−t or smaller than −t, and consider the case where $f\left(x\right)=x^{t+s}$, g(x)=−slnx and $h\left(x\right)=e^{x}$. Then,*

(50)E{Xt}=E{e−slnXXt+s}(51)≥Eexps−lna−1a(X−a)·bt+s+(t+s)bt+s−1(X−b)(52)=es[1−lna]ϕ−sabt+s+(t+s)bt+s−1ψ′−sa−b.

Choosing $b=\psi^{\prime }\left(-s/a\right)$, and changing the optimization variable *a* into α=1/a, we obtain

(53) $$E\left\{X^{t}\right\}\geq \underset{\alpha \geq 0}{\sup}{\left(\alpha e\right)}^{s}\phi \left(-\alpha s\right){\left[\psi^{\prime }\left(-\alpha s\right)\right]}^{t+s}.$$

More specifically, if $X=\sum_{i=1}^{n}Y_{i}$, where $\left\{Y_{i}\right\}$ are Bernoulli i.i.d., with parameter *p*, then $\phi \left(s\right)={\left(pe^{s}+q\right)}^{n}$, where q=1−p. We then obtain

(54) $$E\left\{X^{t}\right\}\geq \underset{\alpha \geq 0}{\sup}{\left(\alpha e\right)}^{s}{\left(pe^{-\alpha s}+q\right)}^{n}\cdot {\left(\frac{npe^{-\alpha s}}{pe^{-\alpha s}+q}\right)}^{t+s}.$$

Selecting α=1/(np), we obtain

(55) $$E\left\{X^{t}\right\}\geq {\left(np\right)}^{t}\cdot \frac{e^{s}{\left(pe^{-s/(np)}+q\right)}^{n}e^{-s(t+s)/(np)}}{{\left(pe^{-s/(np)}+q\right)}^{t+s}}.$$

**Figure 3:**
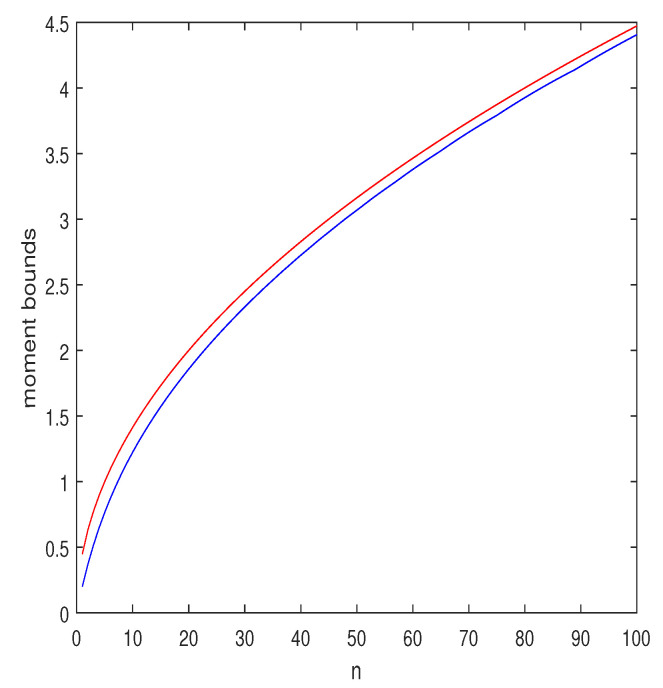
Upper and lower bounds on $E\{\sqrt{\sum_{t=1}^{n}Y_{t}}\}$ as functions of *n*, where $\left\{Y_{t}\right\}$ are i.i.d., Bernoulli (0.2). The red curve is the Jensen upper bound, $\sqrt{np}$, and the blue curve is the proposed lower bound where α is optimized in the range [0,10] and *s* is optimized in the range [0.5,10], both with resolution of 0.01.

The first factor is ${\left(EX\right)}^{t}$. The second factor tends to unity as *n* grows, because $pe^{-s/np}+q\approx p\left(1-s/\left(np\right)\right)+q=1-s/n$, and so, ${\left(pe^{-s/np}+q\right)}^{n}\approx {\left(1-s/n\right)}^{n}\approx e^{-s}$. For t≥1 and t≤0, the function $f\left(x\right)=x^{t}$ is convex, and so, ${\left(EX\right)}^{t}$ is the ordinary Jensen lower bound. In this case, the bound is valuable if the multiplicative factor,

$$\frac{e^{s}{\left(pe^{-s/(np)}+q\right)}^{n}e^{-s(t+s)/(np)}}{{\left(pe^{-s/(np)}+q\right)}^{t+s}},$$

is larger than unity. If 0<t<1, the function $f\left(x\right)=x^{t}$ is concave, and then ${\left(EX\right)}^{t}$ is an upper bound. Of course, the parameter *s* can be optimized, too. Some numerical results for t=0.5 are depicted in Figure 3. As can be seen, the upper and the lower bounds are fairly close.

**Figure 4:**
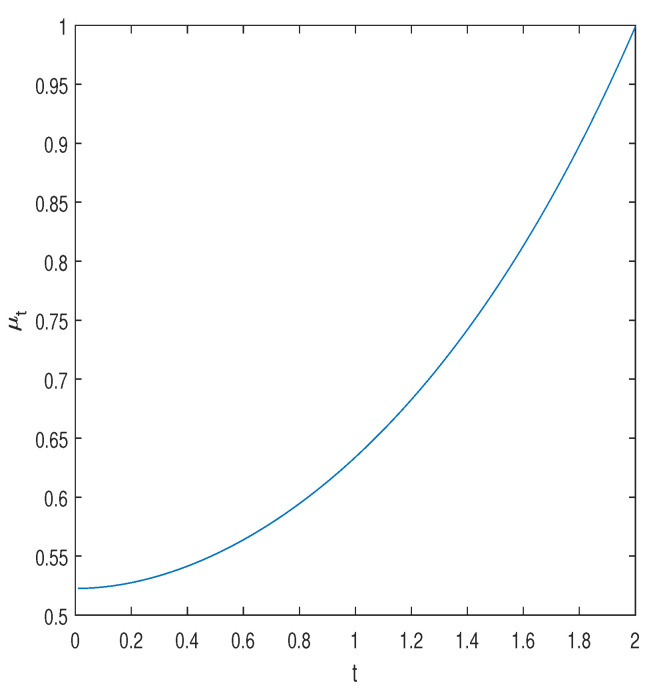
The gap factor, $\mu_{t}$, as a function of *t*. The parameter *s* is optimized in the range [1−t/2,10] with a resolution of 0.001.

Another application of this example is related to estimation theory. Let θ∈R and let $Y_{1},\ldots ,Y_{n}$ be i.i.d., with mean θ and variance $\sigma^{2}$. Consider the *t*-th moment of the estimation error, $E_{\theta }|\frac{1}{n}\sum_{i=1}^{n}Y_{i}{-\theta |}^{t}$. Defining $X={\left(\frac{1}{n}\sum_{i=1}^{n}Y_{i}-\theta \right)}^{2}$, we have

(56) $$\phi \left(s\right)=\frac{1}{\sqrt{1-2s\sigma^{2}/n}};\;\;\;\psi \left(s\right)=-\frac{1}{2}\ln\left(1-\frac{2s\sigma^{2}}{n}\right).$$

and so,

(57) $$\phi \left(-\alpha s\right)=\frac{1}{\sqrt{1+2\alpha s\sigma^{2}/n}};\;\;\;\;\psi^{\prime }\left(-\alpha s\right)=\frac{\sigma^{2}/n}{1+2\alpha s\sigma^{2}/n}.$$

(58)Eθ|1n∑i=1nYi−θ|t=EθXt/2≥(αe)s1+2αsσ2/nσ2/n1+2αsσ2/nt/2+s(59)=σ2nt/2+s·(αe)s(1+2αsσ2/n)(t+1)/2+s.

with either s≥1−t/2 or s≤−t/2. For $\alpha =\zeta n/\sigma^{2}$ (ζ>0 being a constant), we have:

(60) $$E_{\theta }|\frac{1}{n}\sum_{i=1}^{n}Y_{i}-\theta {|}^{t}\geq \frac{\sigma^{t}}{n^{t/2}}\cdot \underset{\zeta >0,\;s>1-t/2}{\sup}\frac{{\left(\zeta e\right)}^{s}}{{\left(1+2\zeta s\right)}^{(t+1)/2+s}}$$

where for t∈[0,2], the first factor, $\sigma^{t}/n^{t/2}$, is the Jensen upper bound. The second factor,

(61) $$\mu_{t}=\underset{\zeta >0,\;s>1-t/2}{\sup}\frac{{\left(\zeta e\right)}^{s}}{{\left(1+2\zeta s\right)}^{(t+1)/2+s}},$$

is the gap between the Jensen upper bound and the proposed lower bound. In Figure 4, we display this factor. The result $\mu_{2}=1$ is expected, because for t=2 and s=0, the calculation is trivially exact. Note that the maximization over ζ, for a given *s*, can be carried out in closed form by equating to zero the partial derivative of $\ln[{\left(\zeta e\right)}^{s}/{\left(1+2\zeta s\right)}^{(t+1)/2+s}]$ with respect to ζ. The optimal ζ turns out to be equal to 1/(t+1) (independently of *s*), and so,

(62) $$\mu_{t}=\underset{s>1-t/2}{\sup}{\left(\frac{t+1}{t+2s+1}\right)}^{(t+1)/2}\cdot {\left(\frac{e}{t+2s+1}\right)}^{s}.$$

Finally, it should be pointed out that this family of Jensen-like bounds opens the door also to lower-bound calculations on the form E{f(X)/g(X)}, where *f* is non-negative convex and *g* is non-negative and concave. Using the fact the identity $1/s=\int_{0}^{\infty }e^{-st}dt$, we have:

(63)Ef(X)g(X)=Ef(X)·∫0∞e−tg(X)dt(64)=∫0∞Ee−tg(X)f(X)dt

and we can apply the same ideas as before to the integrand, having the freedom to optimize the bound parameters with possible dependence on *t*.

## 5. A Product of Two Non-Negative Convex Functions

The last family of Jensen-like bounds that we present in this work is associated with the product of two non-negative convex functions. Let both *f* and *g* be non-negative convex functions of x≥0. Then,

(65)E{f(X)g(X)}≥E{[f(a)+f′(a)(X−a)]·g(X)}(66)=[f(a)−af′(a)]E{g(X)}+f′(a)E{Xg(X))}≥[f(a)−af′(a)]E{[g(b)+g′(b)(X−b)]}+(67)f′(a)E{X[g(c)+g′(c)(X−c)]}f(a)≥af′(a)≥0=[f(a)−af′(a)]·[g(b)−bg′(b)+g′(b)E{X}]+(68)f′(a)[(g(c)−cg′(c))E{X}+g′(c)E{X2}}].

The optimal *b* and *c* are $b^{*}=E\left\{X\right\}$ and $c^{*}=E\left\{X^{2}\right\}/E\left\{X\right\}$, respectively. Thus,

(69) $$E\left\{f\left(X\right)g\left(X\right)\right\}\geq \left[f\left(a\right)-af^{\prime }\left(a\right)\right]\cdot g\left(E\left\{X\right\}\right)+f^{\prime }\left(a\right)E\left\{X\right\}\cdot g\left(\frac{E\{X^{2}\}}{E\{X\}}\right).$$

Let

(70) $$a^{*}=\frac{E\left\{X\right\}\cdot g(E\left\{X^{2}\right\}/E\left\{X\right\})}{g(E\{X\})}$$

and assume that $f\left(a^{*}\right)\geq a^{*}f^{\prime }\left(a^{*}\right)\geq 0$. Then, $a^{*}$ is the optimal value of *a*, which yields

(71) $$E\left\{f\left(X\right)g\left(X\right)\right\}\geq f\left(\frac{E\left\{X\right\}\cdot g(E\left\{X^{2}\right\}/E\left\{X\right\})}{g(E\{X\})}\right)\cdot g\left(E\left\{X\right\}\right).$$

More generally, when *X* and *Y* are two random variables with a joint distribution, the above derivation easily extends to

(72) $$E\left\{f\left(X\right)g\left(Y\right)\right\}\geq f\left(\frac{E\{X\}\cdot g(E\{XY\}/E\{X\})}{g(E\{Y\})}\right)\cdot g\left(E\left\{Y\right\}\right).$$

If *f* and *g* are both concave, rather than convex, then the inequalities are reversed.

**Example 7.**  *Consider again the example of the capacity of the AWGN with a random SNR, c(Z)=ln(1+gZ), and suppose that we wish to bound the variance of c(Z) in order to assess the fluctuations (e.g., for the purpose of bounding the outage probability). Then, obviously,*

(73) $$Var\left\{c\left(Z\right)\right\}=E\left\{c^{2}\left(Z\right)\right\}-{\left[E\left\{c\left(Z\right)\right\}\right]}^{2}=E\left\{\ln^{2}\left(1+gZ\right)\right\}-{\left[E\left\{\ln\left(1+gZ\right)\right\}\right]}^{2}.$$

*To upper bound Var{c(Z)}, we may derive an upper bound to $E\{\ln^{2}\left(1+gZ\right)\}$ and a lower bound to E{ln(1+gZ)}. For the latter, a lower bound was already proposed earlier in Example 5. For the former, we may use the present inequality with the choice f(z)=g(z)=ln(1+gz), which can easily be shown to satisfy the requirements. We then obtain the following upper bound, which depends merely on the first two moments of Z:*

(74) $$E\left\{\ln^{2}\left(1+gZ\right)\right\}\leq \ln\left(1+gE\left\{Z\right\}\right)\cdot \ln\left(1+\frac{gE\left\{Z\right\}\ln(1+gE\left\{Z^{2}\right\}/E\left\{Z\right\})}{\ln(1+gE\{Z\})}\right).$$

*Interestingly, the function $\ln^{2}\left(1+gx\right)$ is neither convex nor concave, yet our approach offers an upper bound, which is fairly easy to calculate provided that one can compute the first two moments of Z.*

## 6. Conclusions

In this work, we have revisited the Jensen inequality on the basis of taking advantage of the freedom to optimize the choice of the supporting line that is tangential to the given convex function. This optimal choice might be different than the ordinary one when the convex function does not stand alone, but it is rather only part of a more complicated expression. This more complicated expression can sometimes be created in an artificial manner, such as in Examples 2, 5 and 6. The resulting bounds depend on either the first two moments of the independent variable, *X*, or on its MGF and its derivative. Both types of moments often lend themselves to relatively easy calculations. The proposed methodology can be used both for improving on the ordinary Jensen inequality (such as in Examples 2 and 4), and for generating lower bounds to expectations of non-convex or even concave (rather than convex) functions (such as in Examples 1, 2, 5 and 7). Several families of Jensen-like inequalities have been derived along with a demonstration of numerical examples with application to information theory. The tightness of the inequalities obtained was also demonstrated in those examples.

## Data Availability

Not applicable.
